# Cell aggregation induces phosphorylation of PECAM-1 and Pyk2 and promotes tumor cell anchorage-independent growth

**DOI:** 10.1186/1476-4598-9-7

**Published:** 2010-01-14

**Authors:** Xing Zhang, Li-hua Xu, Qiang Yu

**Affiliations:** 1 State Key Laboratory of Oncology in Southern China, Sun Yat-sen University Cancer Center, Dongfeng East Road, Guangzhou 510060, China; 2 Department of Pharmacology, Shanghai Institute of Materia Medica, China Academy of Sciences, 555 Zuchongzhi Road, Shanghai 201203, China

## Abstract

**Background:**

Apoptosis caused by inadequate or inappropriate cell-matrix interactions is defined as anoikis. Although transformed cells are known to be anoikis-resistant, the underlying mechanisms have not been well understood. We investigated the mechanisms of anoikis resistance of tumor cells.

**Results:**

We observed that cell aggregation in suspension promoted cell survival and proliferation. We demonstrated a correlation between tumor cell aggregation in suspension and cell growth in soft agar. Analysis of tyrosine kinase-mediated cell survival and growth signaling pathways revealed increased levels of tyrosine-phosphorylation of PECAM-1 and Pyk2 in cell aggregates. We also showed that PECAM-1 and Pyk2 physically interact with each other, and that PECAM-1 carrying a deletion of exons 11-16 could no longer bind to Pyk2. Furthermore, RNA interference-mediated reduction of Pyk2 and PECAM-1 protein levels reduced cell aggregation and inhibited the growth of tumor cells in soft agar.

**Conclusions:**

The data demonstrated that Pyk2 and PECAM-1 were critical mediators of both anchorage-independent growth and anoikis resistance in tumor cells.

## Background

Cell-extracellular matrix (ECM) interactions are essential for survival and growth of normal epithelial cells. In the absence of matrix attachment, these cells have been shown to undergo anoikis, a form of apoptosis [1]. Anoikis is important in maintaining normal cell and tissue homeostasis to ensure a dynamic balance of cell proliferation, differentiation, and apoptosis [2]. Anoikis resistance and anchorage-independence are hallmarks of oncogenic transformation and appear to play an important role in tumor progression and metastasis [3,4].

Previous studies have shown that tumor cells tend to form aggregates in the absence of matrix attachment. The size and number of aggregates have been found to correlate with survival [5,6]. Tumor cells that formed aggregates in suspension cultures were found to exhibit significantly lower levels of apoptosis than single cells, indicating an increased resistance to anoikis. Cell aggregation has also been found to correlate with colony formation in soft agar and tumorigenecity *in vivo *[5-8].

Micrometastases resulting from such tumor cell aggregates are thought to survive within the circulation or bone marrow as small multicellular clusters or spheroids, thereby effecting suppression of anoikis, which is a key property of these cells [9]. In addition, previous research demonstrated that tumor cells cultured in three-dimensional (3-D) aggregates could be used to explain possible mechanisms of drug resistance [10]. The basis of cell aggregation is not well described. Studying the molecular mechanisms mediating cell aggregation could be very important in understanding tumor cell growth and proliferation.

Cell-ECM and cell-cell interactions are mediated by three classes of cell adhesion molecules: cadherins, integrins, and Ig-superfamily proteins. The cadherins are cell-surface proteins that mediate homophilic and calcium-dependent cell-cell adhesions, crucial for structural organization and differentiation of cells [11-14]. Integrins are heterodimeric, cation-dependent cell-membrane adhesion molecules that mediate cell-cell and cell-ECM interactions [15,16]. Integrins play an important role in cell spreading, invasion, and survival.

PECAM-1/CD31 (platelet endothelial cell adhesion molecule-1), expressed on the surface of platelets and leukocytes and at the lateral junctions of endothelial cells, has been implicated in various biological functions, such as leukocyte transmigration, cell migration, angiogenesis, cell signaling, and cell adhesion [17]. Recently, PECAM-1 expression has been found on many tumor cells, such as human brain gliomas, carcinoma of the cervix, lung cancer, and breast cancer [18-26]. However, the significance of PECAM-1 expression in these cells is not fully understood. The relationship between lung-cancer PECAM-1 expression and cell adhesion, proliferation, and migration prompted speculation that this protein may play a role in the formation of tumor cell aggregates.

Pyk2 (Proline-rich tyrosine kinase 2) belongs to the FAK (focal adhesion kinase) family. FAK is activated by the ECM, and it functions in cell motility and adhesion-dependent survival [27]. The molecular structure of Pyk2, its expression pattern, its physical association with paxillin and other cytoskeletal proteins, and its potential roles in multiple signaling pathways suggest that it might play a pivotal role in various cellular events. Pyk2 is involved in several cellular functions, such as adhesion, motility, cell proliferation, apoptosis, and the G_1_-to-S phase transition of the cell cycle [28-31]. Pyk2 also plays a role in the regulation of prostate cell proliferation and its expression may represent a sensitive marker of differentiation of prostate cells [32-34]. While most of these data were obtained from non-transformed epithelial cells, several studies on prostate cancer cells suggest the involvement of Pyk2 activation in invasion processes [32,35]. Moreover, some reports have shown high levels of Pyk2 expression in certain cancer cells, such as breast cancer, lung cancer, and hepatocellular carcinoma, suggesting that it may play a role in cancer cell proliferation, migration, and invasion[36-38]. Therefore, we hypothesized that Pyk2 may play an important role in mediating cell aggregation. Although Pyk2 interacts with many of the same proteins as FAK, the functions of these protein interactions are poorly understood [36,39].

In this study, we investigated the relationship between tumor cell aggregation and growth in suspension cultures. We studied the function of cell aggregates and cell aggregate-mediated signaling pathways. We demonstrated that cell aggregation promotes tumor cell growth in suspension. The ability of tumor cells to form aggregates in polyHEMA suspension correlated with their ability to grow in soft agar. We also report that the Pyk2/PECAM-1 complex is a key signal transducer meditating cell aggregation-generated survival and/or growth signals.

## Methods

### Cell culture

Kidney epithelial cells (MDCK), human lung adenocarcinoma cells (NCI-H1792), human non-small cell lung cancer cells (SK-LU-1), human lung adenocarcinoma cells (A549), and large cell human lung carcinoma cells (NCI-H460) were obtained from American Type Cell Culture Collection (ATCC, Rockville, MD). The cells were cultured in Dulbecco's Modified Eagle Medium (DMEM) supplemented with 10% fetal calf serum (FCS), 100 IU·ml^-1 ^streptomycin, and 100 μg·ml^-1 ^penicillin. HBE4-E6/E7 (human papillomavirus 16E6/E7 transformed normal lung bronchus epithelial cells), obtained from ATCC, were cultured in Keratinocyte Serum-Free Medium (GIBCO, USA) with 0.05 mg·ml^-1 ^bovine pituitary extract, 5 ng·ml^-1 ^recombinant human epithelial growth factor, and 10 ng·ml^-1 ^cholera toxin.

### Soft agar assay

Cells were suspended in 0.3% agar medium (DMEM containing 10% FBS) and then plated on a 0.6% agar base layer at a concentration of 4 × 10^4 ^cells per 60-mm dish. The cells were incubated in a humidified atmosphere (5% CO_2_) at 37°C. The number of colonies that were 50 μm or larger were counted after two weeks.

### Cell aggregation assay

Cells were grown to 70% confluence and then trypsinized. They were then seeded in 60-mm polyHEMA-coated petri dishes at different densities and incubated for different periods of time. The cells were subsequently examined under an inverted phase-contrast microscope, photographed, and counted. The polyHEMA-coated dishes were prepared by coating the plates twice with 2 ml polyHEMA solution [10 mg/ml polyhydroxyethylmethacrylate (Aldrich Chemical Co., Milwaukee, WI) in ethanol] and were dried in a tissue culture hood for 24 h. They were then washed twice with PBS before use.

### DNA extraction and electrophoresis

Cells were lysed in DNA lysis buffer containing 0.5% Triton X-100, 10 mM EDTA (pH 8.0) and 10 mM Tris (pH7.6). The cell lysates were kept on ice for 1 h and then extracted twice with an equal volume of chloroform/phenol, followed by one extraction with chloroform. DNA was precipitated, washed with 70% ethanol, air-dried at room temperature, and resuspended in TE buffer (10 mM Tris·Cl, 1 mM EDTA, pH 7.5). DNA was resolved by electrophoresis in 2% agarose and photographed.

### Flow cytometry analysis

Apoptosis was measured with the Annexin V-FITC Apoptosis Detection KIT (CALBIOCHEM) and analyzed by flow cytometry (FACScan; Becton Dickinson).

### Immunoprecipitation analysis

Cells were washed with PBS and lysed in Triton lysis buffer (2% Triton X-100, 10 mM EGTA, 15 mM HEPES, 145 mM NaCl, 0.1 mM MgCl_2_, 1 mM phenylmethylsulfonyl fluoride, 10 mg·ml^-1 ^aprotinin, 10 mg·ml^-1 ^leupeptin, and 2 mM sodium orthovanadate, pH 7.4) at 4°C for 30 min. Triton-soluble and -insoluble fractions were separated by centrifugation at 15,000 × *g *for 5 min at 4°C. The supernatant was precleared by incubating with 40 μl of protein A-agarose beads for 30 min at 4°C and then centrifuged. Precleared lysates were incubated overnight with PECAM-1 or Pyk2 antibody, and the immune complexes were recovered by incubating with 40 μl of protein-A agarose beads and centrifuging. The immunoprecipitates were washed three times with the Triton lysis buffer and then resolved by 7.5% SDS-PAGE.

### RNAi plasmid construction

pRETRO-SUPER RNA interference constructs were created as described previously [40]. The oligonucleotide sense strand for PYK2 was 5'-gatccccCTGGTCAAATGCACTGTCCttcaagagaGGACAGTGCATTTGACCAG tttttggaaa3', and for PECAM was 5'-gatcccc ACCACTGCAGAGTACCAGCttcaagagaGCTGGTACTCTGCAGTGGT tttttggaaa3'. The 19-nt target sequences are indicated in capital letters.

### Cell transfection and retroviral infection

Ecotropic retroviral supernatants were produced by transfection of packaging cells using calcium-phosphate precipitation method. At 48 h post-transfection, the cell culture medium was filtered through a 0.45 μm filter, 4 μg/ml polybrene was added, and the virus-containing supernatant was used to infect H460 cells. After 6 h of infection, cells were allowed to recover for 24 h in fresh medium. Infected cells were selected with puromycin (2 μg/ml) (Invitrogen, Carlsbad, CA) for 48 h.

### Co-immunoprecipitation of PECAM and Pyk2 in 293T cells

293T cells (1 × 10^6^) were transfected with pcDNA 3.1 empty vector, PECAM FL construct, PECAM Mu (Δ11-16) construct, PYK2 construct, or a combination of these using Lipofectamine Reagent (Invitrogen, Carlsbad, California). The pcDNA/Pyk2 construct was a kind gift from Dr. Mike Schaller (UNC-Chapel Hill). The full-length PECAM-1 and Δ11-16 PECAM-1 (exons 11-16 deleted) constructs were a kind gift from Dr. Steven M. Albelda (Univ. Pennsylvania). After 48 h, cell lysates were prepared in lysis buffer (100 mM Tris, pH 8.0, 100 mM NaCl, 0.5% Nonidet P-40, 1 mM phenylmethylsulfonyl fluoride) and precleared with protein A-Sepharose beads (Amersham Biosciences). Protein lysates (1-mg aliquots) were immunoprecipitated with anti-PECAM-1 antibody (Santa Cruz Biotechnology, Santa Cruz, CA) or anti-Pyk2 antibody (Biosource), followed by immunoblotting with an anti-Pyk2 antibody and anti-PECAM-1 antibody. Immunoreactivity was detected using the Enhanced Chemiluminescence system (ECL, Amersham Pharmacia Biotech, Piscataway. NJ).

### **Immunoblotting**

Cells were washed with PBS and resuspended in lysis buffer (50 mM Tris PH 7.5, 150 mM NaCl, 1%NP40, 0.1%SDS, 1 mM PMSF, 1 mM orthovanadate, 1 mM Na4P2O7,10 mg·ml^-1^ aprotinin, and 10 mg·ml^-1^ leupeptin ) at 4°C for 30 min and centrifuged (13,000 × *g*, 5 min at 4°C). Protein concentrations of the lysates were determined using the Bio-Rad protein assay kit (Bio-Rad Laboratories, CA ). Protein (30 μg) was resolved on SDS-PAGE and transferred onto a nitrocellulose filter. Blocking was carried out for 1 h at room temperature in 5% nonfat milk made in TBS containing 0.05% Tween 20 (TBST). The membrane was then incubated overnight at 4°C in primary antibody (1:1000 dilution) followed by a 1-h incubation with horseradish peroxidase-conjugated goat anti-mouse or anti-rabbit IgG secondary antibody (1:2000 dilution). The 4G10 (anti-phosphotyrosine) antibody was purchased from Millipore, anti-phosphorylated-Pyk2 (pY-881) was purchased from Biosource (Invitrogen), and anti-cleaved caspase-3 (Asp175) antibody was purchased from Cell Signaling. Immunoreactivity was detected using the Enhanced Chemiluminescence system (Amersham).

## Results

### Cell aggregation induces survival and growth signals

**Figure 1:**
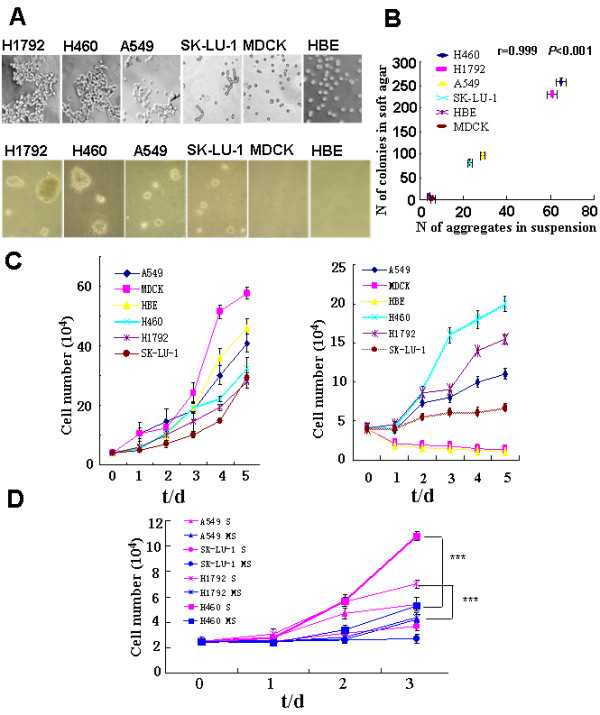
**Cell aggregation correlated with cell growth in suspension culture and in soft agar**. A, Upper panel: Cell aggregation in polyHEMA-coated dishes (× 100). H1792, H460, A549, SK-LU-1, MDCK, and HBE cells were cultured on 60-mm polyHEMA-coated dishes (2.5 × 10^5^/dish) for 8 hours and photographed. Lower panel: Colony-formation of cells in soft agar. 4 × 10^4 ^cells were seeded into 60 mm soft agar plates and incubated for 2 weeks and photographed. B, Correlation between cell aggregation and soft agar growth. The numbers of clonies formed in soft agar after 14 d incubation were plotted against the mean numbers of cell aggregates per 10 fields formed in polyHEMA-coated dishes. Pearson's correlation coefficient was used. N: Number C, Proliferation of tumor cells and normal cells in regular (left) and polyHEMA-coated (right) dishes. Cells as indicated from regular cell culture dishes were plated into regular or polyHEMA-coated dishes (4 × 10^4^/dish) for 1, 2, 3, 4 or 5 d. At the end of each incubation viable cells were counted using trypan blue exclusion. D, Cell aggregation promoted tumor cell proliferation in suspension cultures. 2.5 × 10^4 ^tumor cells as indicated from regular cell culture dishes were plated into polyHEMA-coated or methyl cellulose plates for 1, 2, or 3 d. At the end of each incubation viable cells were counted using trypan blue exclusion. Data from at least three separate experiments were analyzed using Student's t test. "***": *P *< 0.001. S: Suspension culture in polyHEMA-coated dishes MS: Suspension culture in methyl cellulose dishes.

We used several cell culture-based approaches to study the molecular mechanism of tumor cell anoikis resistance, or anchorage-independent survival. Our first strategy was to culture cells in polyHEMA-coated dishes, which prevents cells from attaching to the culture dish and forces the cells to grow in suspension [41,42]. We observed that these tumor cells often formed cell aggregates, and the size and tightness of the aggregates varied depending on the cell line used (Fig. 1A upper). The H1792 and H460 cells formed large and compact aggregates, while the SK-LU-1 and A549 cells formed small and loose aggregates. Most of the MDCK and HBE cells, the non-tumor cell lines, did not form aggregates (Fig. 1A upper).

We analyzed the survival and growth of all of our cell lines in suspension and in soft agar to determine whether cell aggregation played a role in their survival and growth. We found that cell aggregation correlated with soft agar growth (Fig. 1A and 1B) (r = 0.999, *P *< 0.001). Normal MDCK and HBE cells, which did not form aggregates, failed to form colonies in soft agar. The SK-LU-1 cells, which did not form large aggregates in polyHEMA suspension, and the A549 cells, which formed loose aggregates, grew more slowly in soft agar than the H1792 and H460 cells, which formed large, tight aggregates and grew faster in soft agar (Fig. 1A upper and lower). In suspension culture we also found that tumor cells that formed large aggregates, such as H460 and H1792 cells, grew faster than those cells that formed small aggregates, such as SK-LU-1 and A549 cells (Fig. 1C). These results clearly demonstrate that the ability of the tumor cells to form aggregates in polyHEMA suspension not only correlates with the tumor cell growth in polyHEMA suspension but also correlates with their ability to grow in soft agar.

In order to further examine the role of cell aggregation in cell growth, we used methyl cellulose in the culture medium to decrease cell aggregation in suspension. The size of cell aggregates in the H1792 and H460 decreased in the methyl cellulose suspension (MS) cultures compared with that in the polyHEMA suspension (S) cultures. We observed decreased cell growth in the H1792 and H460 cells in methyl cellulose cultures compared to polyHEMA suspension cultures (Fig. 1D). These data suggest that cell aggregation provides growth signals to tumor cells in suspension.

**Figure 2:**
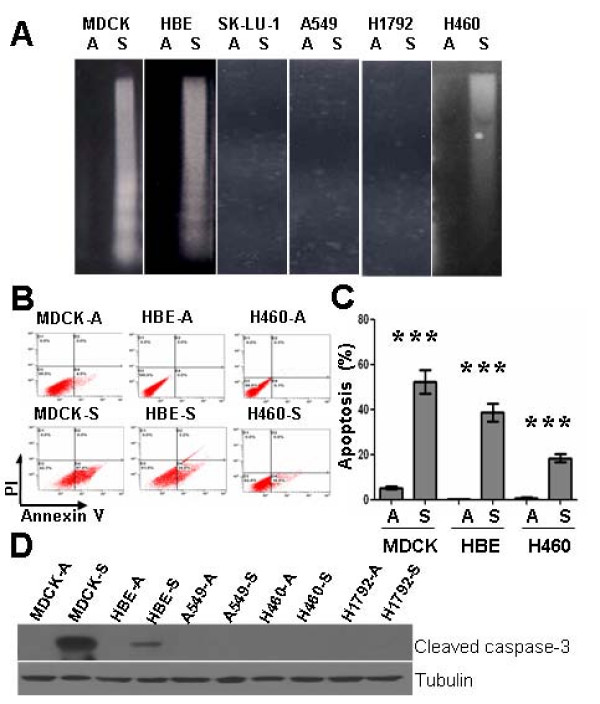
**Detachment from ECM induced apoptosis in normal epithelial MDCK and HBE cells**. A, DNA laddering analysis of cell death. Normal cells and tumor cells from regular cell culture dishes (1 × 10^7 ^) were plated into 100-mm polyHEMA-coated dishes for 24 h and harvested. Cytosolic DNA was extracted and analyzed by agarose gel electrophoresis. B and C Annexin V staining analysis of cell death. 5 × 10^5 ^MDCK, HBE, and H460 cells from regular cell culture dishes were plated into 60-mm polyHEMA-coated dishes for 24 h, harvested, fixed, and assessed by Annexin V/propidium iodide (AV/PI) DNA staining and flow cytometry. B, Percent of early apoptotic cells determined by flow cytometry using AV/PI binding. Quadrant D1: PI/AV +/-, necrotic; D2: PI/AV +/+, late apoptotic/necrotic; D3: PI/AV -/-, live cells; D4: PI/AV -/+, early apoptotic cells. C, Histograms of flow cytometry data. Data from at least three separate experiments were analyzed using Student's t test. "***": *P *< 0.001. A: Attached culture; S: Suspension culture in polyHEMA-coated dishes. D, Immunoblotting analysis of cleaved caspase-3. After the indicated time periods cells were lysed and total protein was extracted separated by SDS-PAGE, and analyzed by immunoblotting with the indicated antibodies. Tubulin was used as a loading control. A: Attached culture; S: Suspension culture in polyHEMA-coated dishes. "***": P < 0.001.

However, we did not observe a correlation between cell aggregation and survival in the case of tumor cells in suspension. The H460 tumor cells, which formed large and tight aggregates in suspension, underwent partial anoikis in suspension (Fig. 2A-C). On the other hand, the SK-LU-1 tumor cells, which did not form large aggregates and grew slowly in suspension (Fig. 1C), were completely resistant to anoikis (Fig. 2A). While aggregation of H460 cells provided adequate signals for cell growth (Fig. 1C), it was not sufficient to protect the cells from undergoing anoikis (Fig. 2A). These data suggest that the signals for tumor cell anchorage-independent survival and growth were distinct.

### Cell aggregation induces tyrosine phosphorylation of PECAM-1 and Pyk2

**Figure 3:**
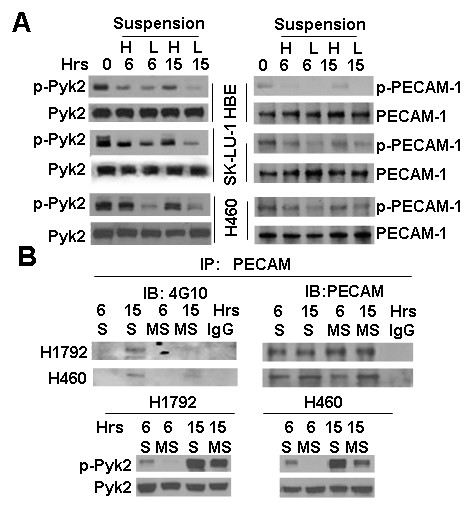
**Induction of phosphorylation of Pyk2 and PECAM by cell aggregation**. A, Phosphorylation of Pyk2 and PECAM in high versus low cell density cultures. Different concentrations of HBE, SK-LU-1, or H460 cells (H: 5.0 × 10^5 ^cells L: 2.5 × 10^5 ^cells) were cultured in 60-mm polyHEMA-coated dishes for 0, 6, or 15 h. Cell lysates were resolved by SDS-PAGE and analyzed by immunoblotting with anti-phospho-Pyk2 (Tyr881), or anti-Pyk2 antibody (left). Equal amounts of whole-cell extracts were immunoprecipitated with anti-PECAM antibody followed by immunoblotting with 4G10 antibody (Right). B. Phosphorylation of Pyk2 and PECAM in regular suspension versus methyl-cellulose suspension cultures. H1792 and H460 cells were cultured in polyHEMA-coated dishes (S) or methyl-cellulose dishes (MS) for 6 or 15 h, harvested, and lysed with RIPA buffer. A, Equal amounts of whole-cell extracts were immunoprecipitated with anti-PECAM antibody followed by immunoblotting with anti-phosphotyrosine 4G10 antibody (Upper). Equal amounts of whole-cell extracts were immunoblotted with anti-pPyk2 (Tyr881) or anti-Pyk2 antibody (Lower).

In order to understand the molecular nature of the cell aggregation-generated survival and growth signals, we analyzed the phosphorylation/activation of PECAM-1 and Pyk2. PECAM protein was immunoprecipitated with PECAM antibody and then immunoblotted with 4G10 antibody (anti-phosphotyrosine monoclonal antibody) to detect tyrosine phosphorylation. Interestingly, we found that the phosphorylation/activation of PECAM-1 and Pyk2 correlated with cell aggregation, although the total protein level of PECAM-1 and Pyk2 were not related to cell aggregation (Fig. 3A, B and Additional file 1 Figure S1). High- and low-density suspension cultures were established in order to study the phosphorylation status of these molecules in relation to cell aggregation. H460, SK-LU-1, and HBE cells formed larger aggregates in high-density cultures compared to low-density cultures. H460 and H1792 formed larger aggregates in polyHEMA cultures compared to methyl cellulose cultures. We observed higher levels of phosphorylated PECAM-1 and Pyk2 in large aggregates (high-density cultures, H) than in small aggregates or single cells (low-density cultures, L) (Fig. 3A)Similarly, higher levels of phosphorylated PECAM-1 and Pyk2 were seen when cells were cultured in large aggregates (polyHEMA suspension culture, S) compared to small aggregates (methyl cellulose suspension culture, MS) (Fig. 3B), suggesting that the phosphorylation/activation of PECAM-1 and Pyk2 correlated with cell aggregation.

### PECAM-1 and Pyk2 knock-down reduce aggregation-mediated cell growth

**Figure 4:**
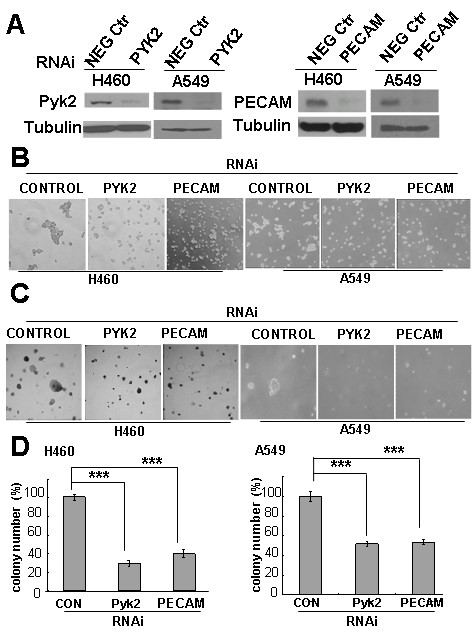
**Knock-down of PECAM-1 or Pyk2 decreased cell aggregation and colony formation in soft agar**. A, H460 (left) and A549 (right) cells were infected with pRetrosuper/Pyk2, pRetrosuper/PECAM-1, or scrambled (negative control) RNAi retrovirus. The cells were then selected with puromycin for 48 h. RNAi-infected cell lysates were analyzed for the level of protein expression by immunoblotting analysis using the antibodies indicated. Anti-tubulin antibody was used to show equal protein loading. B, The morphology of aggregates of H460 cells (left) and A549 (right) infected with PECAM or Pyk2 RNAi vectors. The RNAi-infected cells were selected with puromycin for 48 h. Cells were harvested cultured on 100-mm polyHEMA-coated dishes for 8 h, and photographed by phase-contrast microscope (× 40). C and D Function of PECAM-1 or Pyk2 in promoting soft agar growth of H460 (left) and A549 (right) cells. C. RNAi-infected cells were harvested and plated in soft agar grown for 2 wk at 37°C, and assayed for colony formation. D, Graphical representation of microscopy data expressed as the percentage of colonies. Data from at least three separate experiments were analyzed using Student's t test. "***": *P *< 0.001

The above experiments suggested that the phosphorylation/activatio of PECAM-1 and Pyk2 correlated with cell aggregation. In order to investigate the role of PECAM-1 and Pyk2 in the formation of cell aggregates, we used RNA-interference (RNAi) technology to knock down the expression of PECAM-1 or Pyk2 in NCI-H460 and A549 cells. We found significantly decreased expression levels of the target proteins in the RNAi-infected cells (Fig. 4A).

Cells with knock-down of Pyk2 or PECAM-1 expression showed a decrease in the size and tightness of cell aggregates (Fig. 4B) along with a decreased ability to grow in soft agar (Fig. 4C). PECAM or Pyk2 knock-down cells also formed only about 30% to 50% the number of colonies in soft agar as the control cells (Fig. 4D). Taken together, these data demonstrate that the expression of Pyk2 and PECAM is important in the formation of tumor cell aggregates and could promote the anchorage-independent growth of H460 and A549 cells in soft agar.

### PECAM-1 physically interacted with Pyk2

**Figure 5:**
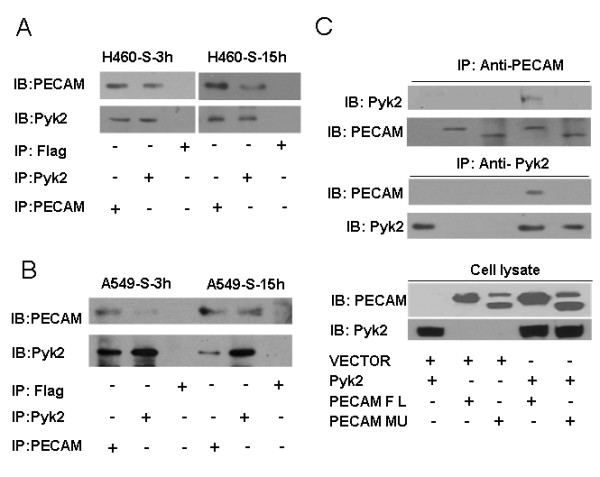
**PECAM-1 interacted with Pyk2 physically *in vivo***. A and B Binding of endogenous PECAM with endogenous Pyk2 in H460 (A) and A549 (B) cells. Confluent H460 and A549 cells from regular cell culture dishes were plated onto 100-mm polyHEMA-coated dishes for 3 or 15 h. Cells were harvested and lysed. Equal amounts of whole-cell extracts were immunoprecipitated with anti-PECAM-1 antibody, anti-Pyk2 antibody, or anti-Flag antibody followed by immunoblotting with anti-Pyk2 antibody or anti-PECAM-1 antibody. C, *In vivo *interaction of Pyk2 and PECAM-1 in 293T cells. 293T cells (1 × 10^6^/100-mm dish) were co-transfected with pCDNA3.1 empty vector, PECAM FL construct, PECAM Mu (Δ11-16) construct, PYK2 construct, or a combination as indicated. After treatment for 48 h, equal amounts of whole-cell extracts were immunoprecipitated with anti-PECAM antibody and immunoblotted for Pyk2 and PECAM (upper) or immunoprecipitated with anti-Pyk2 antibody and immunoblotted for PECAM and Pyk2 (middle). Western blot analysis of equal amounts of cell extracts was also performed in parallel (lower). FL: Full-length; Mu: Mutant (Δ11-16).

Since both Pyk2 and PECAM-1 are important in mediating the formation of tumor cell aggregates and promoting the anchorage-independent growth, we used co-immunoprecipitation to investigate possible interactions between PECAM-1 and Pyk2. We found that PECAM-1 and Pyk2 co-immunoprecipitated in H460 and A549 cells, suggesting an endogenous interaction between these two molecules in these cells (Fig. 5A and 5B).

To further confirm the interaction between Pyk2 and PECAM-1, we co-transfected 293T cells with an expression vector for Pyk2, along with an expression vector for full-length PECAM-1 or PECAM-1 carrying an exon 11-16 deletion. We demonstrated that Pyk2 was co-immunoprecipitated with the full-length version, but not the deletion mutant, of PECAM-1 (Fig. 5C) clearly demonstrating a direct physical interaction between PECAM-1 and Pyk2.

## Discussion

We found that tumor cells formed different sizes of aggregates in suspension cultures and that tumor cells grew slowly when their cell-cell interactions were disrupted. The ability of tumor cells to form aggregates in polyHEMA suspension correlated with their ability to grow in soft agar. Furthermore, the aggregated cells had higher levels of phosphorylated PECAM-1 and Pyk2 than single cells. Inhibition of PECAM-1 or Pyk2 expression using RNA interference impaired cell aggregation and decreased colony-forming ability. We were also able to demonstrate that PECAM-1 and Pyk2 physically interacted with each other. These observations led us to conclude that cell aggregation induced phosphorylation of PECAM-1 and Pyk2 and that the two molecules were key signaling mediators to promote anchorage-independent survival and growth of tumor cells.

Our findings that the ability of tumor cells to form aggregates in polyHEMA suspension correlates with their ability to grow in soft agar are consistent with previous reports. Transformed rat, mouse, hamster, and human cells were found to form aggregates twice as large as those formed by non-transformed cells. The same results have been reported in chemical-transformed rat liver cells and virus-transformed rat and hamster embryo cells [5,43]. Differences in size of cell aggregates have been proposed as novel indicators of malignant transformation [8,44].

We found that the interactions of cell aggregates contribute to inhibition of cell death or anoikis-resistance. While normal cells die upon losing their cell-cell interactions, tumor cells escape anoikis because they either obtain survival signals from increased cell aggregation or acquire bypass mutations [3]. We showed that tumor cells forming large aggregates proliferated faster than tumor cells forming small aggregates. Aggregated cells adhered to one another, were capable of approaching the cellular substratum, and tolerated the three-dimensional stress that affects cell shape, membrane signal transduction, and proliferation. We propose that this could be why cell aggregation promotes cell proliferation. Cell-cell homophilic adhesion or aggregation is also important in tumor cell invasiveness and metastasis [6,9,10,45]. Therefore, identification of key molecules that mediate the cell aggregation-generated cell survival and proliferation signals are important for developing new strategies to treat cancer.

It is known that the cadherin receptor family, integrin receptor family, and activation of EGFR all play a role in promoting anchorage-independent survival and spread of tumor cells. Several studies have shown that cadherins are important for survival signaling, in addition to providing anchorage between neighboring cells [9,46-48]. Recent studies show that FAK (focal adhesion kinase), along with integrin a5β1 (which binds fibronectin), mediates the signaling pathway that results in anchorage of cells to ECM [49,50]. EGFR is important in the survival of colorectal carcinoma cells and confers resistance to anoikis in suspension cultures of normal epidermal keratinocytes and mammary epithelial cells [45,51]. Our data demonstrate that PECAM-1 and Pyk2 are also critical molecules in supporting tumor cell anchorage-independent growth. Future studies are needed to understand the relationship of these molecules.

We found that the phosphorylation of Pyk2 (Y881) correlated with aggregate-dependent cell growth. To our knowledge, we are the first to report a relationship between activated Pyk2 and anchorage-independent growth. Recent reports show that Pyk2 is overexpressed in certain cancer cells, including non-small cell lung cancer, and higher Pyk2 activity is correlated with enhanced cell migration in A549 cells [52,53]. Overexpression of Pyk2 in human HCC cell lines also results in enhanced colony formation and promotes cell proliferation and invasiveness [54]. These data support our finding that activation of Pyk2 promoted formation of cell aggregates and cell proliferation in soft agar, which suggests a possible role of Pyk2 in tumor progression and metastasis.

It is known that phosphorylation of Pyk2 leads to the recruitment of Src-family kinases and the activation of ERKs [54]. However, we found that Pyk2 tyrosine phosphorylation is dissociated from the activation of the MAPK pathway in these aggregation experiments (Additional file 2 Figure S2), suggesting that Pyk2 is involved in other cellular signaling pathways. We previously showed that detachment-induced Src activation in tumor cells contributed to their anoikis resistance. Pyk2, rather than PI3K/Akt or Erk, appears to be the key downstream effecter of Src in mediating the cell survival signals in cell aggregates [55,56]. In future studies, it would be important to investigate the role of the interaction between Src and Pyk2 in tumor cell aggregation.

PECAM-1 has been reported to be expressed in certain carcinoma cells and contributes to tumor cell adhesion [26]. PECAM-1 surface expression plays a role in cancer cell growth and metastasis [57,58]. Interestingly, we found that the phosphorylation of PECAM-1 correlated with aggregate-dependent growth and cell adhesion. Furthermore, knockdown of PECAM-1 expression decreased the size of tumor cell aggregates and their colony-forming ability. These data strongly suggest that activation of PECAM-1 mediates the signaling pathway leading to cell aggregation and growth in suspension.

More interestingly, we found that Pyk2 and PECAM-1 physically interact with each other. This is the first report of an interaction between PECAM-1 and Pyk2. This finding suggests a novel signaling complex that is responsible for cell aggregation and tumor cell anchorage-independent growth. Our future work will investigate the downstream signaling pathways mediated by this interaction.

## Conclusions

Signals generated by cell aggregation are different from cell-matrix interactions. Cell-ECM interactions are mediated mainly by integrin and FAK signals and contribute to cell survival. Tumor cell aggregate formation is mediated by PECAM-1 and Pyk2 signal transduction, leading to cell proliferation. We have identified a novel physical interaction between PECAM-1 and Pyk2, which could play an important role in tumor cell aggregate formation in suspension cultures and in aggregate-dependent proliferation. Our results could be important in providing new insights into the mechanisms of anoikis resistance, tumor progression, and tumor metastasis. It may also provide a new class of targets for the development of new therapeutics in cancer treatment.

## Competing interests

The authors declare that they have no competing interests.

## Authors' contributions

XZ was involved in the design, performed the experiments, and drafted the manuscript. LH X participated in the experiments. QY conceived of the study, contributed to the overall experimental design, and revised the manuscript. All authors read and approved the final manuscript.

## Supplementary Material

Additional file 1 **Expression of Pyk2 and PECAM in non-cancerous and cancer cells**. HBE, MDCK, SK-LU-1, A549, H1792 or H460 cells were cultured on 60-mm regular and polyHEMA-coated dishes for 15 h. Cell lysates were resolved by SDS-PAGE and analyzed by immunoblotting with anti Pyk 2, anti-PECAM, or anti-tubulin antibodies as indicated. Click here for file

Additional file 2 **Phosphorylated level of ERK MAPK in non-cancerous and cancer cells**. HBE, A 549 and H460 cells were cultured on regular and polyHEMA-coated dishes for 15 h. Cell lysates were resolved by SDS-PAGE and analyzed by immunoblotting with anti-phospho-p44/42 ERK MAPK, anti-ERK MAPK or anti-tubulin antibodies as indicated. Click here for file
