# Supplementary material for: Cell aggregation induces phosphorylation of PECAM-1 and Pyk2 and promotes tumor cell anchorage-independent growth
Source: Mol Cancer. 2010 Jan 14;9:7. doi: 10.1186/1476-4598-9-7 (PMC2820017; doi:10.1186/1476-4598-9-7)
Supplement: Additional file 1 — Expression of Pyk2 and PECAM in non-cancerous and cancer cells. HBE, MDCK, SK-LU-1, A549, H1792 or H460 cells were cultured on 60-mm regular and polyHEMA-coated dishes for 15 h. Cell lysates were resolved by SDS-PAGE and analyzed by immunoblotting with anti Pyk 2, anti-PECAM, or anti-tubulin antibodies as indicated. [file 1476-4598-9-7-S1.pdf]

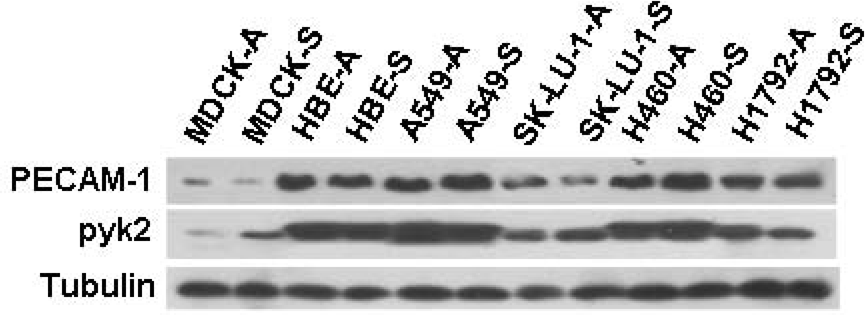
MDCK-A
MDCK-S
HBE-A
HBE-S
A549-A
A549-S
SK-LU-1-A
SK-LU-1-S
H460-A
H460-S
H1792-A
H1792-S
PECAM-1
pyk2
Tubulin
