# Supplementary figures and images for: Cell aggregation induces phosphorylation of PECAM-1 and Pyk2 and promotes tumor cell anchorage-independent growth
Source: Mol Cancer. 2010 Jan 14;9:7. doi: 10.1186/1476-4598-9-7 (PMC2820017; doi:10.1186/1476-4598-9-7)

HBE-A HBE-S A549-A A549-S H460-A H460-S

p-ERK

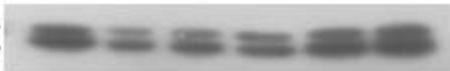

ERK

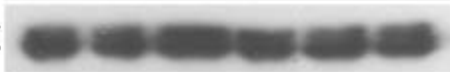

tubulin

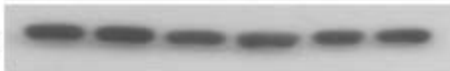

Supplement: Additional file 2 — Phosphorylated level of ERK MAPK in non-cancerous and cancer cells. HBE, A 549 and H460 cells were cultured on regular and polyHEMA-coated dishes for 15 h. Cell lysates were resolved by SDS-PAGE and analyzed by immunoblotting with anti-phospho-p44/42 ERK MAPK, anti-ERK MAPK or anti-tubulin antibodies as indicated. [file 1476-4598-9-7-S2.pdf]
